# Experimental Researches on the Post-Mortem Contractility of the Muscles, with Observations on the Reflex Theory

**Published:** 1847-07

**Authors:** 


Art. I.—*Experimental Researches on the Post-Mortem Contractility of the Muscles, with Observations on the Reflex Theory.* By Bennet Dowler, m.d.—*New York,* 1846. 8vo, pp. 39.

The experimental researches contained in this paper (which is reprinted from the New York Journal of Medicine) are of much interest; as they relate to a phenomenon which we have little opportunity of witnessing on this side of the Atlantic. Many of our readers will remember that, after death from the Asiatic cholera, very marked contractions of the muscles, producing slow but energetic movements of the limbs, not unfrequently presented themselves. These appear to be of frequent occurrence after death by yellow fever; and may occur either spontaneously or in obedience to a stimulus applied to the muscle itself. The following is a description of a well-marked case of the former kind, the subject of which was an Irishman, aged twenty-eight. Upon the cessation of the respiration, the body became quiescent and motionless in every part; but soon afterwards the following series of movements commenced. First he carried his left hand, by a regular motion, to his throat, then to the crown of his head; the right arm followed the same route on the right side; the left hand was then carried back to the throat; thence to the breast, reversing all its original motions; the right hand and arm performed exactly the same motions; in all, eight consecutive, identical, equable motions, in apparently equal times, and with a regularity indicative of volition, though before death he was totally insensible.

Where, however, such movements do not occur spontaneously, they may be excited by simple percussion of the muscle with the hand, with a stick, the flat side of a hatchet, or any other blunt instrument, as in the following case:

"R. C., a Kentuckian, aged 25. In two hours after death, when the arm was extended to an angle of 45° from the trunk, and was struck with my hand, or still better with the side of the hatchet, carried his hand to his epigastrium; but when the arm was extended upon the floor, so as to form a right angle with the body, he slapped himself upon the mouth and nose. The contractility began to decline in the third hour; and by the fourth hour all motions of the limbs ceased, though the pectoral muscles assumed the ridgy or lumpy form when percussed. An hour after death the thigh was moderately contractile. The left heel hung down near the floor; its flexors, after being struck, drew up the heel against the buttock. Five hours after death, the contractility had ceased and rigidity prevailed."

A number of similar experiments, nearly all of them made upon yellow-fever subjects, are related by Dr. Dowler; and in some of them it was proved, by completely separating the limbs from the body, that the influence of the nervous centres is not required for the production of these motions. It is remarkable that in all the cases mentioned, the heat of the body remained at a remarkably high standard for some time after death; and according to Dr. Dowler, it even rose in some instances after the cessation of the respiration. In the case just quoted, the heat continued above the usual standard of health for seven hours after death; only sinking in that time from the extraordinary elevation of 111° to 102°.

We do not see in these phenomena anything which need be a source of perplexity to the physiologist. They obviously result simply from the contractility of the muscle itself, called into action by the mechanical stimulus directly applied to it. Several circumstances appear to favour the manifestation of this property in an unusual degree; especially the high temperature of the body previously to death, and the subsequent retardation of its cooling. This will tend to preserve the vital properties of the muscle; which will manifest themselves the more independently on account of the complete cessation of all nervous influence. The experiments are well worthy of being repeated in this country; for although we do not apprehend that such marked results can be obtained after death from other causes, as Dr. Dowler has encountered in his yellow-fever subjects, still we have little doubt that this post-mortem contractility will be found to be much greater than is usually supposed.

The "Observations on the Reflex Theory," which form the remainder of the pamphlet, are to us utterly incomprehensible. The author declares that he is altogether unable to understand the doctrine of reflex action; and instead of arguing upon it, he puts it to that very worst of all the tests of truth,—ridicule:

> "Whatever agency," he says, "the anterior roots may have when galvanized, they are incomparably inferior to the motor power of the muscle when excited by the hand, the hatchet, or a cane. A hand-, or a hatchet-, or a cane-theory ought to have the preference over the reflex-theory, as the former is not only the most simple but the most scientific, affording a nearer approximation to the known physiological functions of man."

Let Dr. D. explain by his "hand-, hatchet-, or cane-theory," the contraction of the muscles of the *thigh* in a decapitated frog when the *web* is pinched, or the movements of the *legs* of a paraplegic man when the sole of the *foot* is tickled; and the champions of the reflex-theory may then enter the lists with him. But at present he is confusing two sets of phenomena wholly distinct; and his views would make the nervous system of no use whatever.

